# Cannabis Indica

**Published:** 1890-05-24

**Authors:** 


### THERAPEUTICS.

### CANNABIS INDICA.

Dr. J. Russell Reynolds, physician-in-ordinary to Her Majesty's household, has published his experience on the therapeutical uses and toxic effects of Cannabis indica, or Indian hemp. After thirty years' experience of the drug, Dr. Russell Reynolds speaks with a considerable amount of authority. Some of the most markedly valuable results are obtained in certain mental conditions. In senile insomnia, with wandering, Dr. Reynolds has found nothing comparable in utility to Indian hemp extract, in doses of one-quarter to one-third of a grain. In the night restlessness of patients with general paralysis it has proved of eminent utility. But in alcoholic delirium and in melancholia it is not recommended. In painful maladies it is very useful. Neuralgia, periodic or not, often yields to it, even after long durations. Very many victims of migraine have for years kept their sufferings in abeyance by taking hemp at the moment of threatening of an attack. It is also a valuable adjunct to mercury, iodine, or other drugs, but Dr. Reynolds has found hemp almost useless in sciatica, myalgia, pleurodynia and lumbago. The pains that occur only on movement do not, in Dr. Reynolds' experience, receive relief from Indian hemp. It has proved useless also for gastrodynia, enteralgia, and almost all hysterical pains. But it has relieved the lightning pains of the ataxic patient, and also the tingling numbness, anæsthesia, and formication common in the limbs of gouty people. In clonic spasms, whether of the epileptoid or choreoid type, it is useful. But in true chronic epilepsy it is useless. An attack of violent convulsions in an over-fed man, who has had a heavy supper, may be stopped by a full dose of hemp, if they do not cease after clearing the "primæ viæ." It is also a valuable remedy in the nocturnal cramps of old and gouty people In some cases it relieves spasmodic asthma, and is of great service in spasmodic dysmenorrhœa. The drug is one, however, liable to great variations of strength. For practical purposes its active principle has not been separated, and extracts and tinctures cannot be made uniform, because the hemp grown at different seasons and in different places varies. Individuals also differ widely as to their susceptibility to the action of hemp. For an adult, Dr. Reynolds always begins with one-fifth of a grain, and for a child one-tenth. The best form is the tincture, as pills become hard, and the tincture should be taken in drops on sugar or bread. The pharmacopœia tincture contains one grain in twenty minims.

Now it has long been observed that cannabis indica does not affect all persons in a similar manner, and race and climate are also supposed to modify its influence. It has also been observed that women are more powerfully affected by the drug than men, to which Dr. Reynolds does not refer. Its effects have been compared with those of opium. But this comparison can scarcely be made, for the effects are very different. The leaves of the hemp plant are much used in the East, under the name of "bhang," for smoking and to make an infusion of. A resinous exudation from the plant called *churns* is made into sweetmeats. The very small leaves are known as gungah, and gungah has been termed in the Persian "the increaser of pleasure and the assuager of grief." The *hashish* of the Arabs is made from the tender tops of the plant after flowering. It is supposed that the "Nepenthes" of Homer was hemp, and that when "Bright Helen mixed a mirth-inspiring bowl," some part of the hemp plant was used by the fair compoundress. A person under the influence of Indian hemp is often affected much as if he were under alcohol. There is excitement, laughter, afterwards stupidity and insensibility. Before the latter conditions result, a characteristic sensation has been described, as if the brain were boiling over. Some time ago the value of cannabis indica was tested by Fronmuller in 1,000 cases, with success in little more than half that number. Fronmuller said a large dose was required to produce a hypnotic effect, as much as eight grains of the spirituous extract.

It has been mentioned above that the pharmacopœial preparations, the tincture and the extract, differ in strength, and we do not see how this is to be avoided, for the active principle of the plant, whatever it may be, probably differs at various seasons. We are told in the B. P. to use the dried flowering or fruiting tops of the female plants of cannabis sativa, from which the resin has not been removed. We believe, however, that it would be better to use the resinous exudation alone, which would be more likely to afford constant results. We are not aware that this has been tried, although a *cannabin taunas* has been prepared from the hemp plant, which is said to be a useful hypnotic. When we have to deal with a medicine so likely to differ in strength as the pharmacopœial preparations of cannabis indica, and when we recollect that fear, idiosyncrasies, or constitutions, or temperaments, render the action of many drugs uncertain, Dr. Reynolds' caution about commencing with very small doses is not misplaced. It is better to test the patient's tolerance, and gradually increase the dose. For although the toxic effects of cannabis indica are usually recovered from, unpleasant results may be produced, some of which have already been mentioned. Death, however, from even large doses of Indian hemp is very rare. It is often used in India for the purpose of inducing insensibility in order to facilitate theft. The active principle is considered to be a resinous body, *cannabin.* It also contains a volatile oil, and probably a volatile alkaloid. Warden and Waddell are of opinion, from the results of experiments, that both the oil and alkaloid are inert. It is a subject which still requires investigation by the chemists.

